# Variation, Evolution, and Correlation Analysis of C+G Content and Genome or Chromosome Size in Different Kingdoms and Phyla

**DOI:** 10.1371/journal.pone.0088339

**Published:** 2014-02-13

**Authors:** Xiu-Qing Li, Donglei Du

**Affiliations:** 1 Molecular Genetics Laboratory, Potato Research Centre, Agriculture and Agri-Food Canada, Fredericton, New Brunswick, Canada; 2 Quantitative Methods Research Group, Faculty of Business Administration, University of New Brunswick, Fredericton, New Brunswick, Canada; Beijing Institute of Genomics, Chinese Academy of Sciences, China

## Abstract

C+G content (GC content or G+C content) is known to be correlated with genome/chromosome size in bacteria but the relationship for other kingdoms remains unclear. This study analyzed genome size, chromosome size, and base composition in most of the available sequenced genomes in various kingdoms. Genome size tends to increase during evolution in plants and animals, and the same is likely true for bacteria. The genomic C+G contents were found to vary greatly in microorganisms but were quite similar within each animal or plant subkingdom. In animals and plants, the C+G contents are ranked as follows: monocot plants>mammals>non-mammalian animals>dicot plants. The variation in C+G content between chromosomes within species is greater in animals than in plants. The correlation between average chromosome C+G content and chromosome length was found to be positive in Proteobacteria, Actinobacteria (but not in other analyzed bacterial phyla), Ascomycota fungi, and likely also in some plants; negative in some animals, insignificant in two protist phyla, and likely very weak in Archaea. Clearly, correlations between C+G content and chromosome size can be positive, negative, or not significant depending on the kingdoms/groups or species. Different phyla or species exhibit different patterns of correlation between chromosome-size and C+G content. Most chromosomes within a species have a similar pattern of variation in C+G content but outliers are common. The data presented in this study suggest that the C+G content is under genetic control by both *trans*- and *cis*- factors and that the correlation between C+G content and chromosome length can be positive, negative, or not significant in different phyla.

## Introduction

Base composition is a fundamental property of genomes and has a strong influence on gene function and regulation. It is known from Chargaff’s rule [1] that in cellular DNA, the amount of adenine (A) is approximately equal to that of thymine (T) and the amount of cytosine (C) is approximately equal to that of guanine (G). It is also known from Watson and Crick’s pair theory [2] of double-stranded DNA molecules that A pairs with T and C with G. Since the A-T base pair has two hydrogen bonds and the G-C base pair has three hydrogen bonds, the G-C interaction is stronger than A-T. For this reason, C+G content (also called GC content, CG content, or G+C content) is one of the main parameters used to describe DNA base composition.

C+G content can be measured by several methods, including melting–annealing profiles, CsCl gradient buoyant densities, flow cytometry, and genome sequencing, each of which has its advantages and disadvantages. Although C+G content is one of the major factors affecting melting–annealing profiles, the melting–annealing speed of DNA is not 100% correlated with GC content, because the speed is also influenced by the degree of repetition of sequences such as satellite sequences [3]–[5]. For the DNA density bands on the CsCl gradient liquid, it is difficult to achieve a very high resolution in small centrifuge tubes. Flow cytometry measurement is correlated with sequencing data in rice, but the C+G content measured by flow cytometry after staining with DAPI (4′,6-diamidino-2-phenylindole) is consistently higher than the content in the sequencing data [6]. Although the genome-sequencing approach has the highest resolution and is the most precise of all these methods, it is limited by the number of species for which complete genome sequences are available, and its degree of accuracy is affected by the degree of completeness of genome assembly. Shotgun sequencing, together with the assignment of scaffolds to specific chromosomes, can partly address the gap issue. Currently, most of the complete genomes available are from archaeans and bacteria, and a few are from animals and plants. That is likely why the evaluation of base composition in animals has focused mainly on DNA segments and transcribed regions [7]–[9]. In plants, most base compositions are determined on the basis of annealing temperature, and only recently were two dicots and one monocot compared [10]. A general comparison of genomic base composition between major kingdoms (domains of life) is still lacking.

Previous research found that C+G content is positively correlated with chromosome (genome) size in bacteria [11] and some other prokaryotes [12], in keeping with the finding that long coding sequences are usually GC-rich [13], [14]. Since each bacterium usually has a single chromosome, it is unclear whether C+G content correlates positively with chromosome size or genome size and whether C+G content is regulated differently between chromosomes in the same species. One potential scenario is that a higher C+G content makes the chromosome stronger and therefore allows it to be longer. It is unknown whether positive correlation between C+G content and genome or chromosomes size exist in other kingdoms or phyla. However, in a previous study using pooled data for 1 plant, 4 animals, 3 protists, and 7 fungi, no significant correlation were found between the genomic GC contents and genome sizes [15]. It is possible that no correlations were found between the 15 genome sizes and the 15 C+G contents concerned because different mechanisms operate in different kingdoms. To determine whether C+G content is correlated with genome size or chromosome size, it is necessary to analyze many more species and compare the species within each kingdom and subkingdom. Since complete or nearly complete chromosome sequences are available for fungi, protists and some higher eukaryotes, we can examine the correlation between C+G contents and chromosome lengths for those particular species.

We speculate that C+G content and genome size may or may not be positively correlated depending on the kingdom or phylum, but that the species in each subkingdom share similar pattern for the relationship between chromosome length and C+G contents, likely because of shared evolutionary history, similar functional needs, and similar molecular mechanisms. In the present study, we analyzed the base compositions, particularly the C+G contents, of nearly all major kingdoms and phyla by using complete and nearly complete genomes from the GenBank, RefSeq, EMBL, DDBJ, and PDB databases obtained through searches of National Center for Biotechnology Information (NCBI) (http://www.ncbi.nlm.nih.gov/nucleotide/).

## Results

### General Description of Genome Size and C+G Content Variation at the Kingdom and Subkingdom Levels

**Figure 1:**
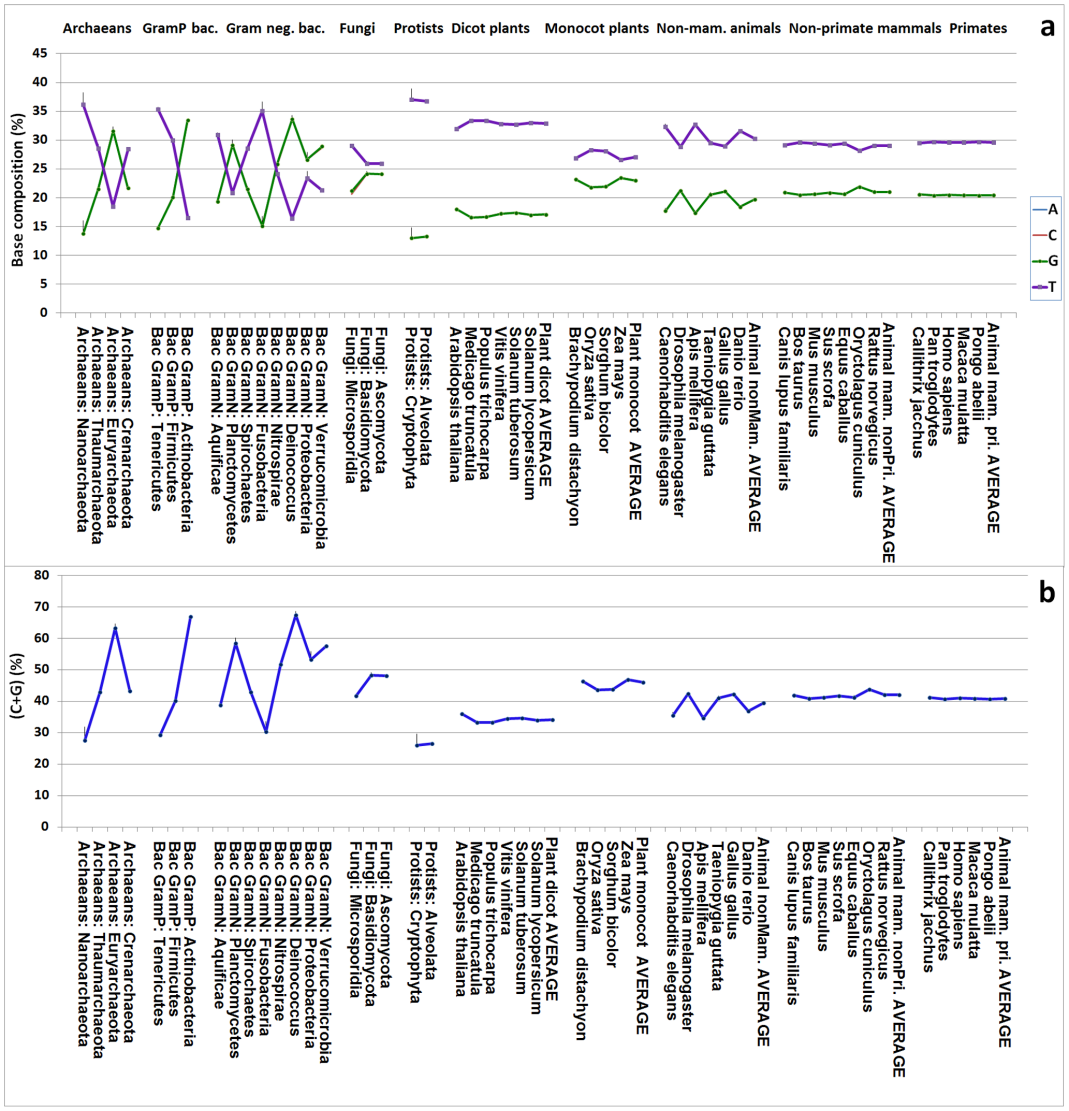
The base compositions and C+G contents of different kingdoms and large groups. The information is provided by phylum for archaeans, gram-positive bacteria, gram-negative bacteria, fungi, and protists and by species for dicot plants, monocot plants, non-mammalian animals, non-primate mammalian animals, and primate animals. Note that in panel (**a**), the A and T contents essentially overlap and are indistinguishable, and a similar situation exist for the C and G contents.

The average genome sizes for the six kingdoms/subkingdoms that were analyzed are listed here in order from smallest to largest: Archaea (1.86 Mb)<Bacteria (2.61 Mb) <protists (3.16 Mb)<fungi (18.44 Mb) <plants (538.84 Mb)<animals (1,837.44 Mb) (Table 1; Figure 1). Microorganisms and higher organisms showed very different patterns of C+G content when the genomes were ranked by size within each large phylogenetic group (Table 1; Figure 1). The genome-level average C+G contents were ranked according to the percentage of the total C+G nucleotides in the total genomic nucleotides of the kingdom or subkingdom, with the results being as follows: protists (26.42%) <plants (41.10%)<animals (41.20%) <Archaea (44.88%)<fungi (47.96%)<bacteria (50.76%) (Table 1).

**Table 1:** Average genome size and average genomic C+G content in different kingdoms and subkingdoms.

Kingdom or subkingdom	Number of genomes	Average genome size (Mb)	Average genome C+G (%)
**Kingdoms:**			
Archaeans	58	1.86	44.88
Bacteria	430	2.61	50.76
Fungi	11	18.44	47.96
Protists	21	3.16	26.42
Plants	10	538.84	41.10
Animals	18	1,837.44	41.20
**Subkingdoms:**			
Gram-positive bacteria	184	2.53	49.40
Gram-negative bacteria	246	2.67	51.71
Dicot plants	6	368.78	34.15
Monocot plants	4	793.93	45.93
Non-mammalian animals	6	674.06	39.44
Non-primate mammalian animals	7	2,190.40	41.97
Primate animals	5	2,739.35	40.85

The protists for which genome size and C+G content were analyzed in this study are from only two phyla, Alveolata and Cryptophyta. It is unclear at this stage whether the genome size and average C+G content (25.7%) obtained is representative of all protists. However, the order shown above for other kingdoms should be very reliable because of the relatively large numbers of species in each kingdom. Although eukaryote species have multiple chromosomes, prokaryotes usually (except three archaeal species) have just one.

In terms of average genome size, we obtained the following ranking: Gram-positive bacteria (2.53 Mb)<Gram-negative bacteria (2.67 Mb); dicot plants (368.78 Mb)<monocot plants (793.93 Mb); and non-mammalian animals (674.06 Mb)<non-primate mammalian animals (2,190.40 Mb) <primate animals (2,739.35 Mb) (Table 1). These results appear to point to an evolutionary increase in genome size.

The ranking of the average genomic C+G content is very similar to that of average genome size in the different subkingdoms of bacteria, plants, and animals. The order found for genome C+G content is as follows: Gram-positive bacteria<Gram-negative bacteria; dicot plants<monocot plants; non-mammalian animals<mammalian animals (Table 1). In higher organisms, the C+G content was lowest in dicot plants and highest in monocot plants, with animals having an intermediate content (Figure 1).

The C+G content, showed considerable variation within each kingdom among microorganisms (Figure 1), was relatively similar among species of dicot plants and monocot plants, and highly similar among species of mammalian animals (Figure 1). With regard to subkingdoms of plants and animals, the C+G content was dramatically different between dicot and monocot plants and also clearly different between non-mammalian animals and mammalian animals, although the difference between non-primates mammals and primates was relatively minor (Figure 1).

### Genome Size and C+G Content Statistical Distribution and Normal Distribution Probability

**Figure 2:**
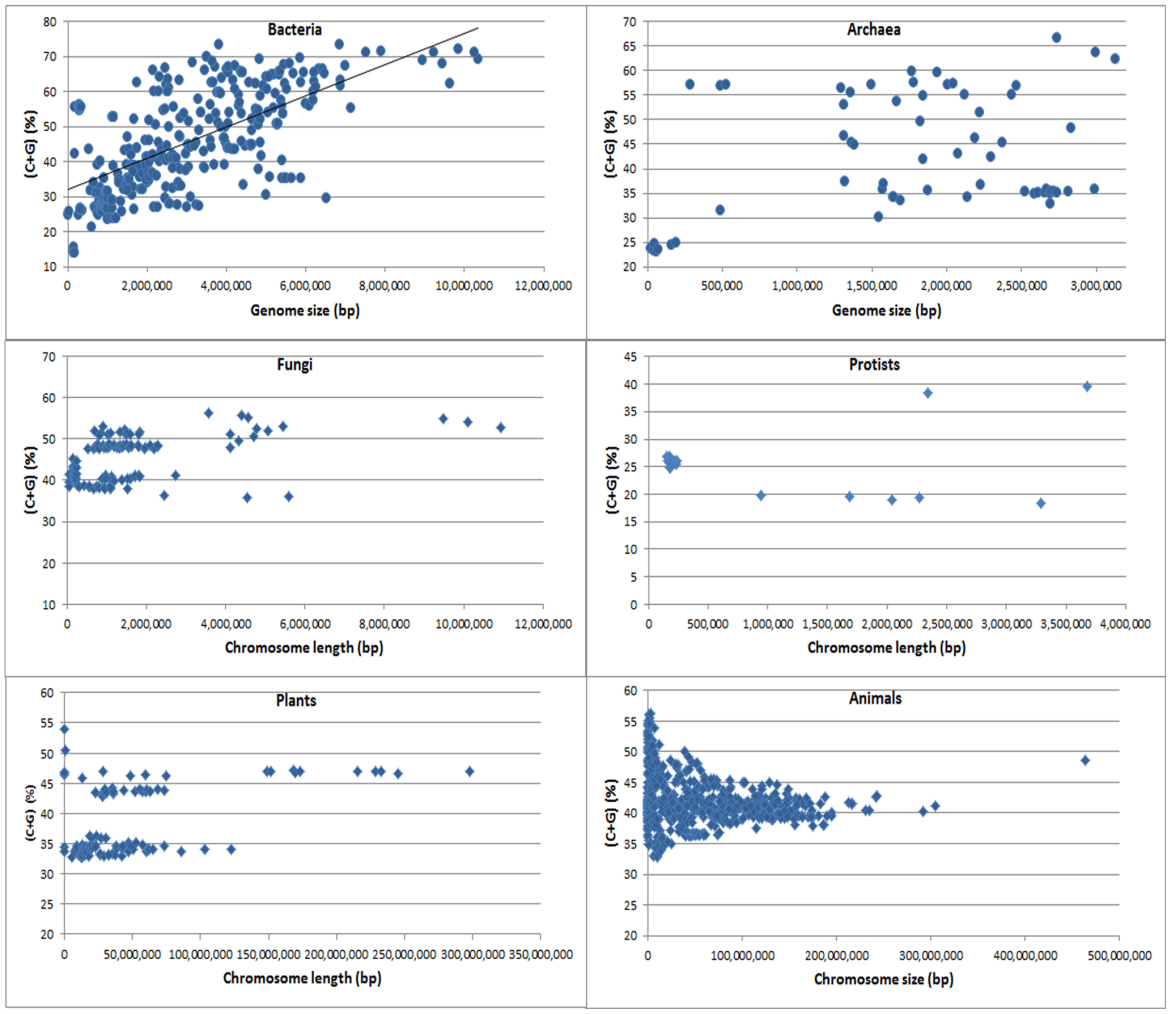
Distribution of values of chromosome size and C+G content in each kingdom of living organisms. The chromosome number for bacteria, archaea, fungi, protists, plants, and animals is 430, 61, 139, 21, 115, and 565, respectively. Note that the C+G content and chromosome or genome size in bacteria is positively correlated but there is no such a correlation or linear relationship in other kingdoms.

When C+G content was plotted against genome size, the distribution of the actual chromosome lengths and C+G contents within each kingdom was found to be very scattered (Figure 2). A clear positive correlation between C+G content and chromosome length was observed, as well as some outliers, in bacteria (Figure 2). In the other kingdoms (Archaea, Fungi, Protists, Plants, and Animals), no linear correlation was found between the C+G content and chromosome length (Figure 2).

**Figure 3:**
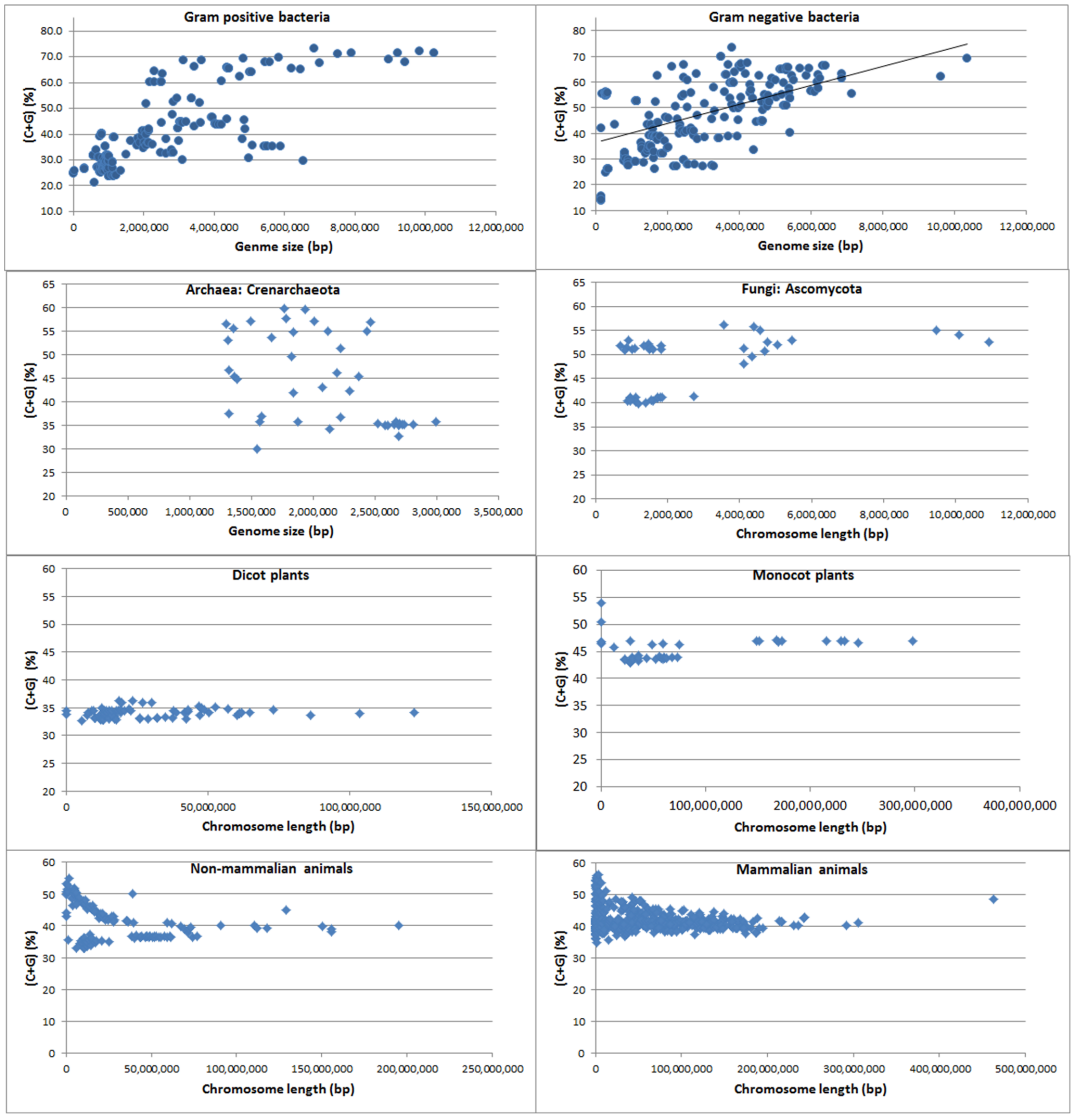
Distribution of values of chromosome size and the C+G content in each subkingdom. The chromosome number for Gram-positive bacteria, Gram-negative bacteria, Crenarchaeota, and Ascomycota is 184, 246, 42, and 61, respectively. The chromosome or large scaffold number for dicot plants, monocot plants, non-mammalian animals, and mammalian animals is 74, 41, 120, and 445, respectively. Note that C+G content and chromosome or genome size in both Gram-positive and Gram-negative bacteria are positively correlated but there is linear relationship in other subkingdoms. Note that in Crenarchaeota (archaea) there are two groups in the distribution. The lower right corner group is Sulfolobus species. There is no correlation between C+G content and genome size within each of the two distribution group of Crenarchaeota.

Like the kingdoms, the subkingdoms did not show a simple linear correlation between C+G content and chromosome size (Figure 3). Gram-positive bacteria and Gram-negative bacteria appear to show clear positive correlations between C+G content and chromosome size (Figure 3). Again, the C+G content and chromosome length data did not show a simple linear relationship, which suggests that the Pearson correlation coefficient model does not apply in this situation.

**Figure 4:**
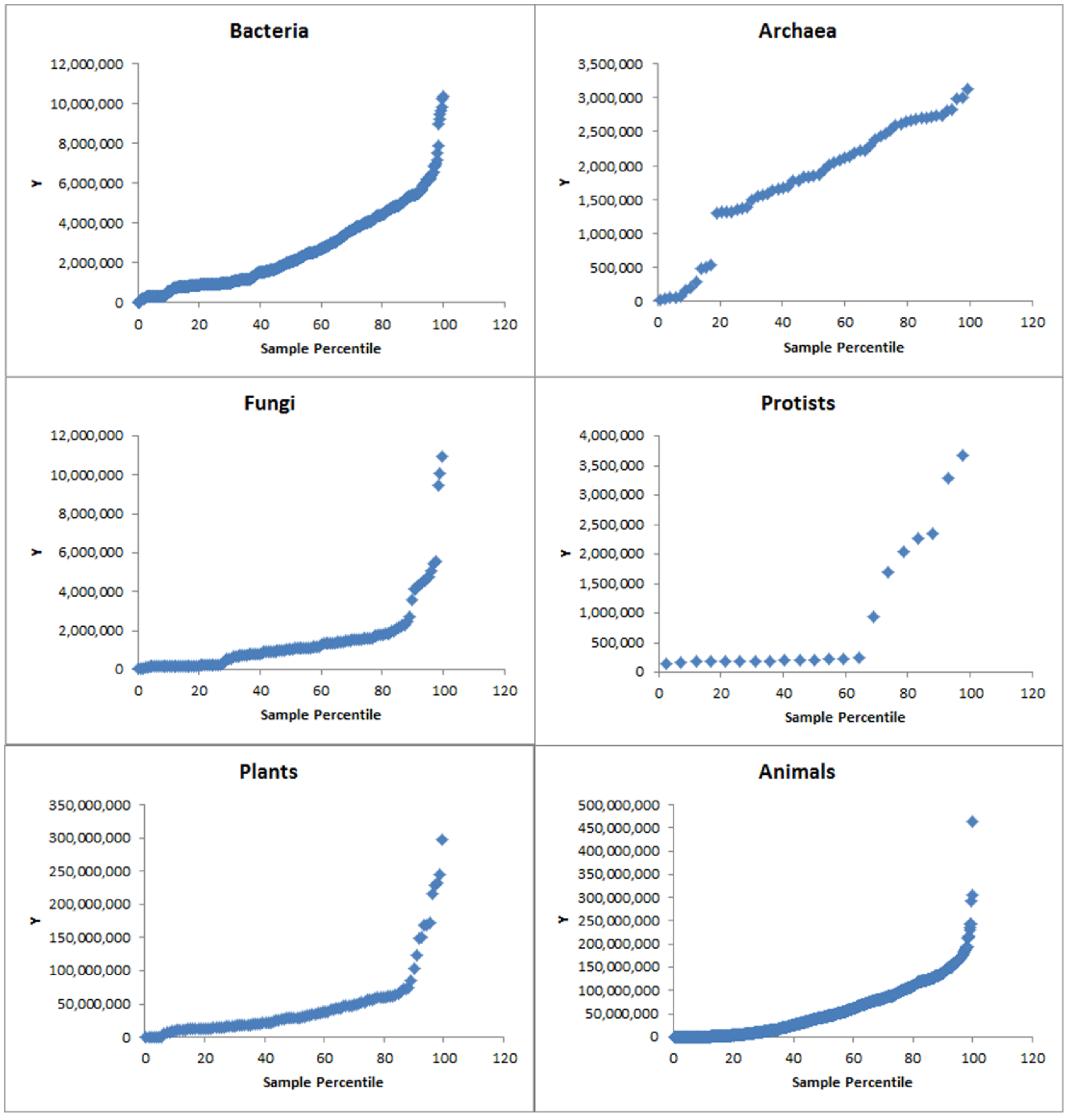
Normal probability plots of genome/chromosome size (bp, the Y-axis). Note that the large genomes/chromosomes (on the right of each plot) do not fit a normal distribution.

**Figure 5:**
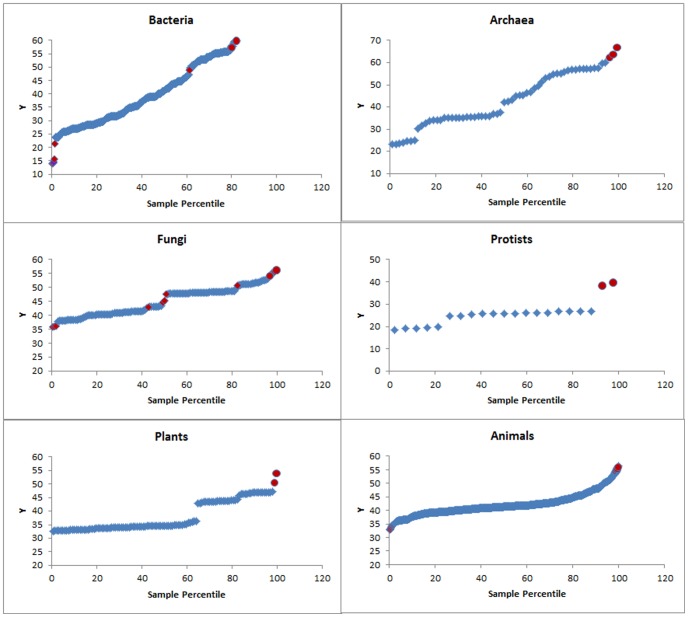
Normal probability plots of C+G contents (%, the Y-axis). Note that the lowest and the highest C+G contents (on the left and right, respectively, of each plot) do not fit a normal distribution. Red dots: outliers.

The probability distribution plots of C+G content and chromosome length for different kingdoms showed that none of the six kingdoms were well modelled by the normal distribution regardless of chromosome length (Figure 4) or C+G content (Figure 5). Interestingly, in each kingdom, the probability plot showed that most chromosomes fit normal distribution fairly well but the longest chromosomes clearly do not fit (Figure 4). Also, in each kingdom, with regard to the chromosome C+G content, the majority of chromosomes fit an approximately normal distribution (despite partitioning into more than one group in some cases); the lowest and highest C+G contents were clearly outliers (Figure 5).

When all the chromosomes within each subkingdom were pooled and plotted together, average chromosome C+G content and chromosome sequence length showed the following patterns: positive linear correlation for Gram-positive bacteria, Gram-negative bacteria, and fungi, as well as dicot and monocot plants; negative correlation for non-mammalian animals and primate animals; and no significant correlations for Archaea and non-primate mammalian animals (Table 2). When all animal chromosomes were pooled and analyzed together, a slight negative correlation was found between C+G content and chromosome size (Table 2). A positive correlation was found between C+G content and chromosome size (Table 2) for pooled dicot and monocot plant chromosomes.

**Table 2:** Correlation between chromosome size and chromosome C+G content in different kingdoms and subkingdoms.

Kingdom or subkingdom	Number of chromosomes	Average chromosome size (Mb)^a^	Average chromosome C+G (%)	Spearman correlation between chr size and C+G (*R*)
**Kingdoms:**				
Archaeans	61	1.77	43.09	0.21 NS
Bacteria	430	2.61	43.71	0.61 **
Fungi	139	1.46	45.09	0.46 **
Protists	21	0.90	25.63	−0.29 NS
Plants	115	46,86	38.11	0.39 **
Animals	565	58,54	42.06	−0.19 **
**Subkingdoms:**				
Gram-positive bacteria	184	2.53	40.05	0.75 **
Gram-negative bacteria	246	2.67	46.45	0.50 **
Dicot plants	74	29.90	34.07	0.26 *
Monocot plants	41	77.46	45.41	0.32 *
Non-mammalian animals	120	33.70	41.55	−0.43 **
Non-primate mammalian animals	165	92.93	42.03	−0.09 NS
Primate animals	280	48.91	42.29	−0.20 **
Mammals (pooled)				−0.14 **

a The smaller chromosome/scaffold size in primate animals relative to non-primate mammalian animals is likely due to the incompleteness of genome assembly.

*, **, and NS: Significant (P<0.05), highly significant (P<0.01) and not significant (P>0.05) according to the “algorithm AS 89” test (see Methods for more details).

**Figure 6:**
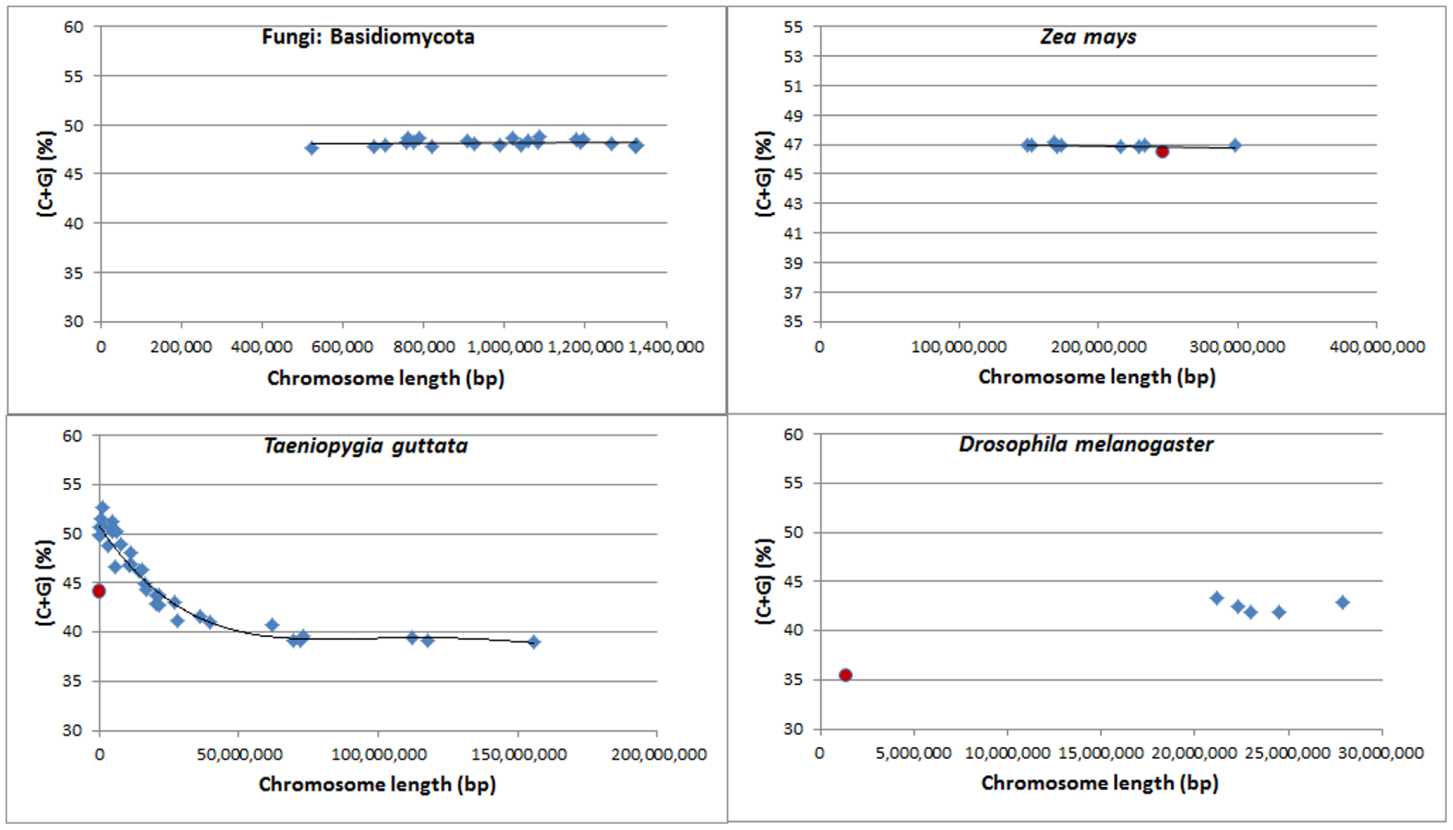
Distribution of C+G contents and genome/chromosome sizes showing some outliers. Blue diamonds: genome or chromosomes which appear to be normally distributed. Red round dots: outliers.

At the small phylum or species level, in some cases such as in the Basidiomycota (fungi), the C+G content and chromosome length were found to be normally distributed, but no clear-cut correlations were found (Figure 6). Although most chromosomes fit normal distribution, outliers were frequently found, such as in *Zea mays*, *Drosophila melanogaster*, and *Taeniopygia guttata* (Figure 6).

In summary, Pearson models of linear regression were not suitable for calculating correlation coefficients. We decided to use the Spearman model to calculate the monotonic relationship between C+G content and chromosome size for each kingdom (Table 2), subkingdom (Table 2), and large species groups (Table 3).

**Table 3:** Genome sizes, C+G contents, and correlations between genome or chromosome size and C+G content in different phyla and species analyzed.

Kingdom, phylum, or species^a^	No. of chrs^b^	Average genome size^c^	Genome C+G content^d^ (%)	Genome (A+T)/(C+G) ratio	Spearman correlation (chr size and C+G)^e^ (R)
**Archaeans**					
Crenarchaeota	42	2,072,521	43.2	1.32	−0.52^f^^ **^
Euryarchaeota	6	3,388,400	63.3	0.58	0.60 NS
Nanoarchaeota	8	135,584	27.6	2.62	0.62 NS
Thaumarchaeota	5	1,970,970	43.0	1.33	0.50 NS
**Bacteria: Gram-positive**					
Actinobacteria	37	5,162,387	67.0	0.49	0.73 **
Firmicutes	59	3,360,830	40.1	1.49	0.03 NS
Tenericutes	88	857,257	29.3	2.41	0.11 NS
**Bacteria: Gram-negative**					
Aquificae	14	1,638,805	38.7	1.59	−0.06 NS
Deinococcus	7	3,064,306	67.3	0.49	0.18 NS
Fusobacteria	7	2,499,267	30.2	2.31	0.43 NS
Nitrospirae	4	2,821,645	51.6	0.94	0.80 NS
Planctomycetes	33	1,892,222	58.4	0.71	0.33 NS
Proteobacteria	130	3,195,588	53.3	0.88	0.84 **
Spirochaetes	47	1,937,957	43.0	1.32	0.27 NS
Verrucomicrobia	4	3,664,906	57.5	0.73	0.80 NS
**Fungi**					
Ascomycota	61	17,487,539	48.2	1.08	0.47 **
Basidiomycota	42	18,762,089	48.2	1.08	0.05 NS
Microsporidia	36	2,218,723	41.8	1.39	0.14 NS
**Protists**					
Alveolata	7	8,125,950	26.5	2.77	0.14 NS
Cryptophyta	14	540,299	26.0	2.84	−0.27 NS
**Dicot plants**					
*Arabidopsis thaliana*	5	118,960,141	36	1.78	−0.20 NS
*Medicago truncatula*	8	245,176,270	33.2	2.02	−0.31 NS
*Populus trichocarpa*	20	260,960,130	33.3	2	−0.06 NS
*Solanum lycopersicum* g	10	718,969,627	34	1.95	0.49 NS
*Solanum tuberosum*	12	578,393,875	34.7	1.88	0.64 *
*Vitis vinifera*	19	290,237,009	34.4	1.9	0.37 NS
**Monocot plants**					
*Brachypodium distachyon*	10	224,012,348	46.3	1.16	−0.47 NS
*Oryza sativa*	12	370,733,456	43.6	1.3	0.47 NS
*Sorghum bicolor*	9	549,132,399	43.8	1.28	0.32 NS
*Zea mays*	10	2,031,824,535	46.9	1.13	−0.44 NS
**Animals: non-mammalian**					
*Apis mellifera*	14	164,585,183	34.7	1.88	0.27 NS
*Caenorhabditis elegans*	6	100,269,912	35.4	1.82	−0.54 NS
*Danio rerio*	26	1,474,835,229	36.8	1.72	0.27 NS
*Drosophila melanogaster*	6	120,290,946	42.4	1.36	0.26 NS
*Gallus gallus*	33	1,169,412,079	42.1	1.37	−0.81 **
*Taeniopygia guttata*	35	1,014,970,640	41.0	1.44	−0.91 **
**Animals: non-primate mammalian**					
*Bos taurus*	9	1,068,912,767	40.9	1.44	0.02 NS
*Canis lupus familiaris*	11	898,613,247	41.8	1.39	−0.08 NS
*Equus caballus*	32	2,335,454,483	41.2	1.42	−0.10 NS
*Mus musculus*	26	1,135,801,574	41.2	1.42	0.21 NS
*Oryctolagus cuniculus*	23	2,603,978,348	43.7	1.29	−0.50 **
*Rattus norvegicus*	45	5,058,733,204	42	1.38	−0.23 NS
*Sus scrofa*	19	2,231,281,778	41.7	1.4	0.01 NS
**Animals: primate**					
*Callithrix jacchus*	23	2,177,235,585	41.1	1.43	−0.31 NS
*Homo sapiens*	190	2,804,441,965	40.9	1.44	−0.12 NS
*Macaca mulatta*	23	2,871,002,222	40.9	1.45	−0.48 *
*Pan troglodytes*	25	2,752,354,403	40.7	1.46	−0.56 **
*Pongo abelii*	26	3,093,520,335	40.7	1.46	−0.43 **

a From archaeans to protists: by phylum or large group; from plants to animals: by species.

b The number of chromosomes. Because archaeans and bacteria (except for the three archaean species in the Euryarchaeota group, as shown in Figure 7) have one chromosome per cell, the number equals the genome number (except for the Euryarchaeota group of archaeans. Euryarchaeota. The average genome size in Euryarchaeota was 3,388,400 bp).

c From archaeans to protists: average genome size of all the species in the phylum or large group; from animals to plants: genome size of the species.

d The overall C+G content when all the genomes or chromosomes were pooled.

e The size and C+G content of each chromosome were calculated first, and then sizes and C+G contents were used to calculate the Spearman R value.

f R  = 0.04 after removing *Sulfolobus* spp.

g *Solanum lycopersicum* (tomato) chromosomes 8 and 12 were omitted from this calculation because NCBI did not have the complete sequences of those chromosomes at the time of downloading.

*, **, and NS: Significant (P<0.05), highly significant (P<0.01) and not significant (P>0.05) according to the “algorithm AS 89” test (see Methods for more details).

### General Comparative Analysis of Chromosome Size and C+G Content at the Kingdom and Phylum Levels

C+G contents were ranked (in terms of the average C+G content per chromosome or large scaffold) among kingdoms, yielding the following results: protists <plants<animals<Archaea<bacteria<fungi (Table 2). In microorganisms, chromosome size or length in terms of nucleotide number was ranked from smallest to largest: protists>fungi>archaeans>Gram-positive bacteria>Gram-negative bacteria (Table 2). Animal and plant chromosome sizes were less precise because some chromosomes were not complete; however, the assembled chromosomes were already very large, with average sizes ranging from 29.90 Mb in dicot plants to 92.93 Mb in non-primate mammalian animals. These were likely large enough to be used in estimating the approximate C+G contents of the chromosomes (Table 1).

### Chromosome Analysis Within Archaeans

Archaeal genome size varied considerably. The average genome sizes of the four phyla Nanoarchaeota, Thaumarchaeota, Crenarchaeota and Euryarchaeota were 0.14, 1.97, 2.07, and 3.39 Mb, respectively (Table 3). The average chromosome size in Euryarchaeota was 1.69 Mb, because each of its genomes had two chromosomes (Table 3). In archaeans, chromosome size was equal to genome size in all species except in the phylum Euryarchaeota in which there were two chromosomes. The genomes in the phylum Nanoarchaeota phylum were the smallest and they had very low C+G contents (27.6%), with an (A+T)/(C+G) ratio of 2.62 (Table 3). The phylum Euryarchaeota had the highest C+G content (63.3% on average) and the lowest (A+T)/(C+G) ratio (0.58) (Table 3).

For the pooled chromosomes of all the archaeal species, the Spearman correlation between C+G content and chromosome length was weak (*R*  = 0.21) and not significant (Table 2). When each subkingdom was analyzed separately, a highly significant Spearman *R* value (−0.52) was obtained for the Crenarchaeota (Table 3). However, when we plotted the data, two found two distinct groups (Figure 3). The genomes located in the right lower corner were from three species of Sulfolobus. When we analyzed the two groups separately, the Spearman *R* value was for the Sulfolobus group was 0.01, and the value for the major Crenarchaeota group was 0.04 (Figure 3). Therefore, there was no correlation between C+G content and genome size in the two subgroups of Crenarchaeota.

Three species from the phylum Euryarchaeota (*Halorubrum lacusprofundi*, *Haloarcula hispanica*, and *Haloarcula marismortui*) each had one larger chromosome (2.7–3.1 Mb, called Chromosome 1) and one smaller chromosome (288–525 kb, called Chromosome 2). Both of these chromosomes had the highest C+G contents among archean species (Table 3; Figure 1); their contents were much higher than those of the species of Thaumarchaeota and Crenarchaeota. The smaller chromosomes had relatively lower C+G contents than did the larger chromosomes in all three species. Chromosome length and C+G content showed a positive correlation when all chromosomes in these three species were analyzed together (Figure 7a,b). However, this correlation does not necessarily mean that C+G content actually increased when chromosome size increased. We verified this by conducting separate analyses of Chromosome 1 and Chromosome 2 from all the Euryarchaeota species and found that C+G content decreased when the size of Chromosome 1 increased; a similar pattern was also observed for Chromosome 2 (Figure 7c,d).

**Figure 7:**
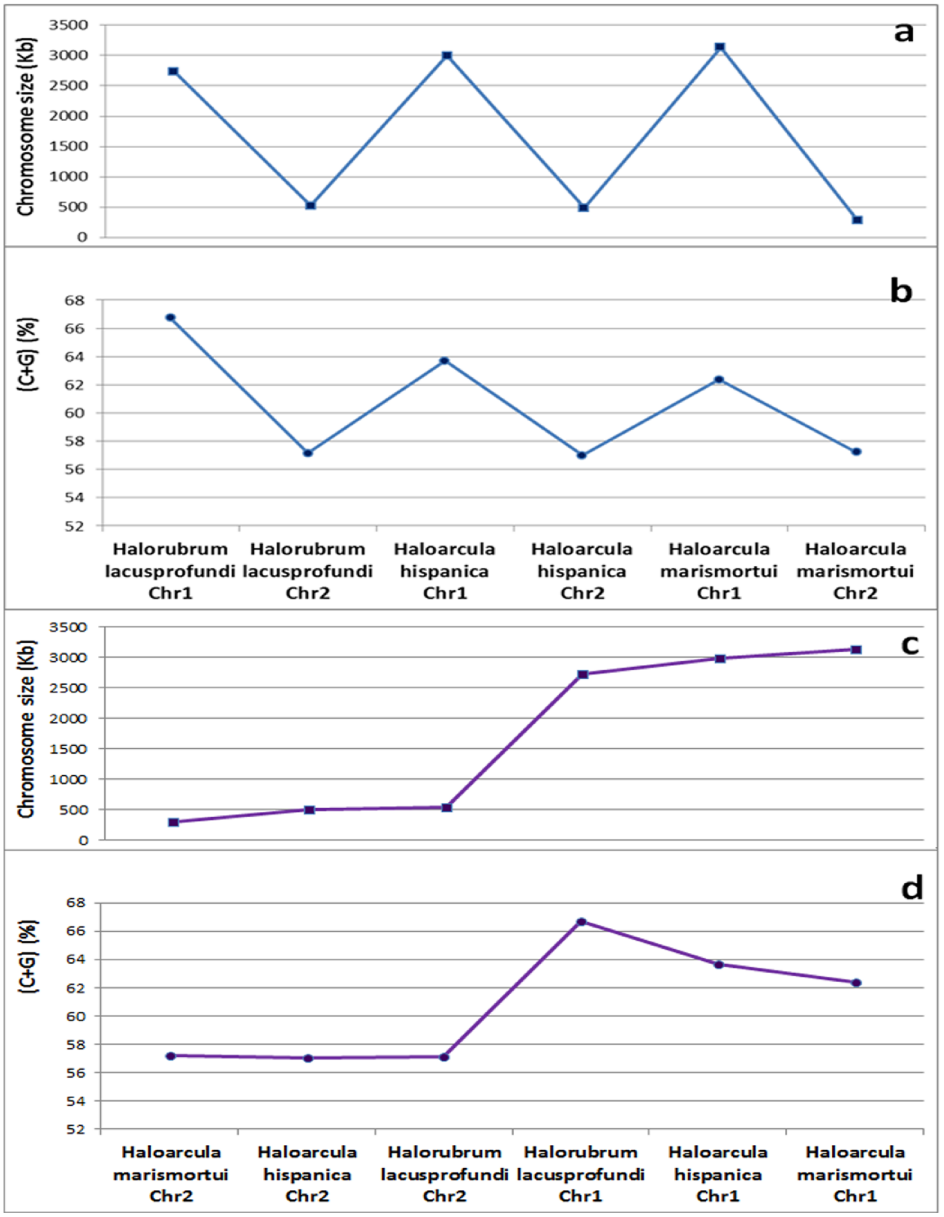
The chromosome sizes and C+G contents of three Archaea species, which each have one large chromosome (Chr1) and one small chromosome (Chr2). The three species (*Haloarcula hispanica*, *Haloarcula marismortui*, and *Halorubrum lacusprofundi*) belong to the Euryarchaeota. Note that although the C+G contents appear to be correlated with chromosome sizes in (**a**) and (**b**), the C+G contents are actually negatively correlated with chromosome size within each type of chromosome (Chr1 and Chr2), as shown in (**c**) and (**d**).

From the above analysis, it is more plausible to conclude that there was likely no moderate or strong correlation between C+G content and genome size in the 61 archaeal genomes analyzed in this study. This issue may need to be revisited once a much larger number of archaeal species have been sequenced, with a focus on whether there is a negative correlation between C+G content and chromosome size in Euryarchaeota.

### Chromosome Analysis Within Gram-positive Bacteria

In total, 184 genomes of Gram-positive bacteria were analyzed and the general correlation between C+G content and genome size was found to be very strong (*R*  = 0.75; Table 2 for correlation; Figure 3 for distribution). The genomes were from three phyla–Tenericutes, Firmicutes, and Actinobacteria–with average genome sizes of 0.86, 3.36, and 5.16 Mb, respectively, and average C+G contents of 29.3%, 40.1%, and 67.0%, respectively. In terms of the average genome size of Gram-positive bacteria, there was a clear increase in C+G content with increasing genome size (Figure 1). When all the chromosomes were ranked from smallest to largest (figure not shown), the first 88 genomes (small) were from the phylum Tenericutes and the remaining genomes were from the other two phyla. Although the Tenericutes genome showed an approximately fivefold increase in size among its species, there was no tendency toward increasing C+G content (*R*  = 0.11; Table 3). No correlation was found for Firmicutes, either (Table 3). The only positive correlation between C+G content and genome size was found among the species of the Actinobacteria group (Table 3). Among the Gram-positive bacteria, there were at least two evolutionary patterns: genome size was strongly and positively associated with genomic C+G content in the Actinobacteria group but no correlation was found in the other two phyla (Table 3).

### Chromosome Analysis Within Gram-negative Bacteria

Eight phyla or large groups of Gram-negative bacteria, namely (in order of average genome size) Aquificae, Planctomycetes, Spirochaetes, Fusobacteria, Nitrospirae, Deinococcus, Proteobacteria, and Verrucomicrobia, were analyzed (Table 3). When all the genomes of the Gram-negative bacteria regardless of phylum were ranked from smallest to largest, there was clearly a tendency toward increasing C+G content with increasing genome size (R  = 0.50) (Table 2; Figure 3). When genome-size ranking was done for each phylum or large phylogenetic group, marked increases in C+G content and genome size were observed in the phylum Proteobacteria. While this relationship may also characterize the phyla Nitrospirae and Verrucomicrobia, it may not be significant owing to the small number of genomes. However, no such relationship was found for Aquificae, Spirochaetes, Planctomycetes and Deinococcus, and the results for Fusobacteria were not clear. The C+G contents ranked from only 14.55% in *Carsonella ruddii* (isolate Thao2000, CP003544, 157.5 kb) to 69.19% in *Myxococcus stipitatus* (CP004025, 10,350 kb). This corresponds to a 67-fold difference in genome size and a 4.8-fold difference in C+G content between the two species. The C+G content increase appears to have continued at the same rate during the evolution of this phylum Proteobacteria (data not shown). These results suggest that genome size and C+G content have coevolved in some bacterial phyla but not in others.

### Chromosome Analysis Within Fungi

The three fungal phyla (Microsporidia, Basidiomycota, and Ascomycota) showed phylum-level C+G content increases in association with genome size increases (Figure 1). There was a moderate positive correlation between C+G content increase and genome size increase in Ascomycota (*R*  = 0.47) but not in Microsporidia or Basidiomycota (Table 3).

### Chromosome Analysis Within Protists

Among the two large groups or phyla (Alveolata and Cryptophyta) of protists analyzed, there was a very large difference in genome size, but the C+G contents were quite similar. There was no correlation between C+G content and chromosome length in these two phyla of protists (Table 3). When we pooled and plotted all the chromosomes, we found a weak negative correlation but with at least two outliers corresponding to exceptionally high C+G contents (Figure 2). The correlation between C+G content and chromosome length in protists should be re-examined after more protist genomes are sequenced.

### Chromosome Analysis Within Dicot Plants

Six dicot plants, namely *Arabidopsis thaliana*, *Medicago truncatula*, *Populus trichocarpa*, *Vitis vinifera*, *Solanum tuberosum*, and *Solanum lycopersicum*, were analyzed (Table 2; Figure 1). The average genome size was 0.37 Mb when the unanchored scaffolds were not counted. In the dicot group, *A. thaliana* had the smallest genome (119.0 Mb), *S. lycopersicum* the largest (719.0 Mb), and *S. tuberosum* (Group Phureja) the second largest (578.4 Mb) when the unanchored scaffolds were not counted. Given that the current draft genome (PGSC_DM_v3_2.1.10) of *S. tuberosum* (Group Phureja) has 11% unanchored sequences, actual chromosome size may be a few percent larger. All the dicot plant species had much smaller C+G contents than did the mammalian animals (Figure 1).

**Figure 8:**
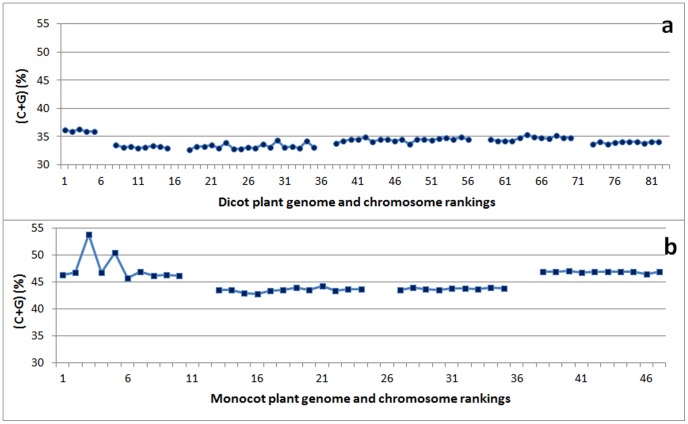
The C+G contents (%) per chromosome in dicot and monocot plants, ranked by genome size among species and by chromosome size within each species. Panel (**a**) shows dicot plants, namely (in order from left to right) *Arabidopsis thaliana*, *Medicago truncatula*, *Populus trichocarpa*, *Vitis vinifera*, *Solanum tuberosum*, and *Solanum lycopersicum*. For *S. lycopersicum*, chromosomes 6 and 8 were analyzed together as one file, and chromosome 10 and 12 also as one file because they were together in the downloaded files. Panel (**b**) shows monocot plants, namely (in order from left to right): *Brachypodium distachyon*, *Oryza sativa*, *Sorghum bicolor*, and *Zea mays*. Note that there is very little variation in C+G content between chromosomes within each dicot or monocot species, with the exception of *B. distachyon*.

Genomic C+G content was remarkably similar in these plants, with exception of *A. thaliana* (Figure 8a). When all the plant chromosomes were pooled together, there was a positive but relatively weak correlation between C+G content and genome size (R  = 0.39; Table 2). When each species was analyzed separately, potato (*S. tuberosum*) was found to have a strong positive correlation between C+G content and chromosome length (*R*  = 64; Table 3). Tomato (*S. lycopersicum*) showed a moderate correlation (*R*  = 0.49) but it was not significant (Table 3). The other plant species did not show a significant correlation. Since the complete genome sequence is not available for either the potato or the tomato, the correlation between C+G content and chromosome size should be re-examined in future to determining whether the correlation is indeed positive, once the genome assembly in these two closely related species has been enhanced further.

### Chromosome Analysis Within Monocot Plants (Cereals)

Four monocot plants were analyzed (Figure 1). These cereals, namely *Brachypodium distachyon*, *Oryza sativa*, *Sorghum bicolor*, and *Zea mays*, were ranked in order of genome size from smallest to largest (Table 3). The average genome size was 0.79 Gb when the unanchored scaffolds were not counted. In the monocot group, *B. distachyon* had the smallest genome (0.22 Gb) and *Z. mays* the largest (2.03 Gb) when the unanchored scaffolds were not counted. The C+G contents in these monocot plants, despite variation among species (Figure 8b), were much higher than those in the dicot plants and were also higher than those in the non-mammalian, non-primate mammalian, and primate animals (Figure 1).

When we pooled all the monocot plant chromosomes together, we found a weak positive correlation between C+G content and genome or chromosome size (*R*  = 0.32; Table 2). When each species was analyzed separately, none of the species showed a significant correlation between the C+G content and chromosome length (Table 3). Although the C+G content in *B. distachyon* differed among the chromosomes (Figure 8b), very similar C+G contents were found among the chromosomes within species in other three cereals, regardless of chromosome size (Figure 8b). The correlation between C+G content and chromosomes size was moderately positive in *O. sativa* but moderately negative in *B. distachyon* and *Zea mays* (Table 3).

### Chromosome Analysis Within Non-mammalian Animals

The six non-mammalian animal species with complete or nearly complete genome sequences had an average genome size of 674 Mb and an average genome C+G content of 49.4% (Table 1). There were clear differences in genome size among the six genomes. *Caenorhabditis elegans* had the smallest genome (100.27 Mb) and *Danio rerio* the largest (1,474.84 Mb, or 1.47 Gb).

For these species, at the genome level, there was no clear correlation between C+G content and average genome size (Figure 1). However, when all the 120 chromosomes of these six animals were pooled, a negative correlation was found between C+G content and chromosome size (*R* = −0.43; Table 2). Interestingly, two species, *Taeniopygia guttata* and *Gallus gallus*, showed a very strong negative correlation between C+G content and chromosome size (*R*  = 0.91 and *R*  = 0.81, respectively; Table 3). Despite the general negative correlation between C+G content and chromosome size in *G. gallus*, two large chromosomes actually had quite high C+G contents. This suggests that chromosome-level *cis*-regulation may exist, in addition to the overall cell-level regulation (Figure S1).

### Chromosome Analysis Within Non-primate Mammalian Animals

This analysis encompassed seven non-primate mammalian species whose sequence scaffolds are clearly assigned to specific chromosomes, even though the contiging is often incomplete (Figure 1). The average genome size was 2.19 Gb. There were clear-cut differences in genome size among the seven genomes. *Canis lupus familiaris* had the smallest genome (0.90 Gb) and *Rattus norvegicus* the largest (5.06 Gb). However, there was no clear correlation between C+G content and genome size (Figure 1). Within species, C+G content and chromosome size showed a moderate negative correlation (*R* = −0.50) in *Oryctolagus cuniculus* but there was no significant correlation in the other non-primate mammalian animals (Table 3).

**Figure 9:**
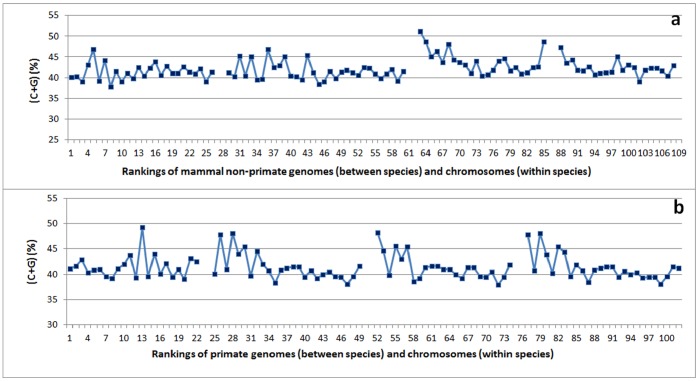
The C+G content (%) per chromosome in mammals, ranked by genome size among species and by chromosome size within each species, from smallest to largest. Panel (**a**) shows non-primate mammalian animals, namely (in order from left to right) *Mus musculus*, *Equus caballus*, *Oryctolagus cuniculus*, and *Rattus norvegicus*. Panel (**b**) shows primate animals, namely (in order from left to right) *Callithrix jacchus*, *Pan troglodytes*, *Macaca mulatta*, and *Pongo abelii*. Note that the patterns are quite similar among the last three species of primate animals.

In Figure 9a, the genome sizes of four nearly complete genomes are ranked from smallest to largest among species, and the chromosome sizes are arranged from smallest to largest within each species. *Mus musculus* and *Equus caballus*, two species with relatively small genomes, showed similar pattern of C+G content variation when chromosome sizes were arranged from smallest to largest (Figure 9a, first two species). The larger chromosomes within each species tended to have smaller C+G contents. Interestingly, *Oryctolagus cuniculus* and *R. norvegicus*, two species with relatively large genome sizes, had very similar patterns of C+G content among their chromosomes: both the largest and smallest chromosomes within each species tended to have larger C+G contents than the chromosomes of intermediate size (Figure 9a, last two species).

### Chromosome Analysis Within Primate Animals

Five primate species (Table 2) were selected for analysis because they had nearly complete genome sequences with clear grouping of scaffolds for each chromosome (Figure 1). Their genomes were all very large, with an overall average genome size of 2.74 Gb. The average C+G content in primates (40.9%) was higher than that in non-mammalian animals (39.44%) but lower than that in non-primate mammals (41.97%) (Table 1). The genome size differences among the five primates were less clear-cut than those found among the non-primate animals. *Callithrix jacchus* had the smallest genome (2.18 Gb) and *Pongo abelii* the largest (3.09 Gb) when the unanchored (unlocated) scaffolds were not counted. The C+G contents of these primate animals were remarkably similar and quite consistent relative to those of other groups of living organisms (Figure 1).

Within each species, C+G content and chromosome size showed a consistently negative correlation, which reached a high level of significance in *Pan troglodytes*, *Macaca mulatta*, and *Pongo abelii* (Table 3). Four species (*C. jacchus*, *P. troglodytes*, *M. mulatta*, and *P. abelii*) had the most clearly defined chromosomes or pseudomolecules within the primate group. When these four genomes were ranked from smallest to largest among species and the chromosome sizes were arranged from smallest to largest within each species (Figure 9b), three species (*Pan troglodytes*, *M. mulatta*, and *Pongo abelii*) had very similar C+G content variation patterns (Figure 9b). The smallest chromosome group within each species tended to have the largest C+G content, and the largest two or three chromosomes also had relatively high C+G contents in comparison with the intermediate chromosomes (Figure 9b). The C+G content variation patterns for these species produced an imperfect U shape in Figure9b. These corresponding patterns in primates showed some similarity to, but a more pronounced U shape than the patterns in the non-primates *Oryctolagus cuniculus* and *Rattus norvegicus* (Figure 9a, last two species). This finding may provide insight into the evolutionary relationship between primates and some non-primates. Further research is required to understand the similarity in the relationship between C+G variation patterns and chromosome sizes.

## Discussion

### Positive, Negative or No Correlation Between C+G Content and Genome Size or Chromosome Length Depending on Kingdoms or Phyla

In this general analysis, in which all chromosomes within each large group were pooled, we found the following relationship between C+G content and chromosome size: strong positive correlation in Gram-positive bacteria and Gram-negative bacteria; strong negative correlation in some animal species (among chromosomes within species in *Taeniopygia guttata* and *Gallus gallus*); and some positive correlations in fungi and plants (Tables 2 and 3). In plants, given the general difference in C+G contents between dicot and monocot plants, a separate analysis is required within each large group.

Most of the plant and animals species in this genome study were also used in our previous analysis of RNA poly(A) site selection [16]. Even through fungi, protists, plants and animals have very different genomic C+G contents, as showed in the present study, the poly(A) tail starting position of the poly(A) site is usually an adenosine in every species [16]. However, the preference for C over G at the mRNA poly(A) tail attachment position is very different in animals and plants: there is almost no difference between C and G in animals, a moderate bias toward C versus G in dicot plants, and a strong preference for C over G in monocot plants [16]. A large-scale analysis is required in order to obtain an overview of genome characteristics (or genome biology traits).

In our analysis, we found that C+G content and genome or chromosome size were positively correlated, negatively correlated, or not correlated in a kingdom-, phylum-, or species-dependent manner. In bacteria, the correlation was positive in 2 of the 11 phyla (within currently available genomes), a finding that may explain the positive correlation previously reported for bacteria [11], [12]. However, we found a lack of correlation for 9 of 11 bacterial phyla and an existence of negative correlation between chromosome size and C+G contents in animal species (Table 3).

Unlike the situation for prokaryotes and fungi, in which C+G content and chromosome size were either positively correlated or not correlated, negative correlations were found in animal species (Table 3). Similarly, gene direction evolved from mainly same-direction neighbours in archaeans, bacteria and protozoa (protists) to opposite-direction neighbours in fungi, chlorophyta (protists) and some animals [17]. These findings point to the need for large-scale analyses of different kingdoms in genome biology research. Both gene direction and the correlation between C+G content and genome/chromosome size provide evidence of evolutionary change associated with increasing biological complexity.

### Independent Emergence of Strong Correlation Between C+G Content and Genome Size or Chromosome Length During Evolution

Bacteria and Archaea diverged likely since a very early cellular life form appeared on Earth. The first phylogenetically distinct branches for their evolutionary descent from a common ancestor have not been identified. However, in some phylogenetic trees [18], bacterial phyla Aquificae and Deinococcusare are among early bacterial branches in terms of the relative branching order, and archean phylum Crenarchaeota also likely branched out earlier than most other archaeal phyla [19]. A quite different tree was derived recently based on an alignment of 24 marker genes; however FastTree was used and the bootstrap support value was very low [20]. Therefore, controversy still exists regarding which clades represent the earliest branches from the common ancestors of the Bacteria or Archaea. If Aquificae and Deinococcusare represent two of the basal branches on the phylogenetic tree for bacteria, the data in the present study may suggest that the early evolutionary branches in bacteria lack any strong correlation between C+G content and genome size. Taken together, Aquificae bacteria, archaeans, and fungi all lacked a strong correlation between C+G content and genome or chromosome size. Therefore, it is likely that the genomes of the basal branches on the tree of life were characterized by a lack of strong correlation between C+G content and genome size. The strong correlations found, such as the strong positive correlation in Actinobacteria and Proteobacteria and the strong negative correlation in some animals, likely emerged independently later after the divergence from their last common ancestor.

### Different Genetic Controls for the Gram Staining Trait and the Correlation Between C+G Content and Genome Size in Bacteria

In Gram-positive bacteria, the C+G content and genome size showed a very strong positive correlation in the phylum Actinobacteria but there was no detectable correlation in the phyla Firmicutes and Tenericutes (Table 3). In Gram-negative bacteria, the positive correlation between C+G content and genome size was very strong in Proteobacteria but almost no correlation was found in Aquificae (Table 3). It is known that most Gram-positive bacteria fall in the same large clade on the bacterial phylogenetic tree [21]. The results clearly indicate that the correlation between C+G content and genome size and the Gram staining reaction trait are two different genetically controlled traits.

The phyla Aquificae and Deinococcus are phylogenetically distinct from most other Gram-negative bacteria, and Deinococcus is located on a branch that is dominated by Gram-positive dominated bacteria [21]. In a recent phylogenetic study [20], Aquificae and Deinococcus were grouped together on a large branch that is located between Gram-negative and Gram-positive bacteria. These two bacterial phyla can be in the same situation with regard to the absence of correlation between C+G content and genome size, regardless of whether this pattern has existed since they emerged or is the result of mutation. It provides further evidence that the correlation between C+G content and genome size and the Gram staining colour are two separate traits and controlled by different genes.

### Evidence of Both *Cis*- and *Trans*-regulation of Genome Size and C+G Content

The probability plot of genome size in Figure 4 suggests that most genomes or chromosomes fit a normal or nearly normal distribution but the largest genomes and chromosomes stand apart. The results indicate that most chromosome sizes fall within the normal distribution range, likely under a common unknown *trans-*acting regulation that operate on these chromosomes at the cellular level, but some large chromosomes cannot be explained by the common regulation. It is likely that the largest chromosomes were created through some specific events, which may include processes such as chromosome fusion, translocation, *cis*-acting transposition and *cis*-acting gene amplification.

The probability plot of C+G content in Figure 5 suggests that most genomes have the same normal or nearly normal distribution for C+G content. The existence of outliers is very clear in the case of *Drosophila melanogaster* (Figure 6), *Gallus gallus* (Figure S1), *Zea mays* (Figure 6) and T*aeniopygia guttata* (Figure 6). In *Drosophila melanogaster*, the high similarity (41.8% to 43.3%) of C+G content in Chromosomes 2L, 2R, 3L, and X points to a cellular-level influence on C+G content. Chromosome 4 (C+G content: 35.5%) in *Drosophila melanogaster* is an exception, which provides evidence for the existence of local GC-poor mechanism (i.e., *cis*-factor) specifically applying to this chromosome.

Other evidence in support of *cis*- regulation for the variation of C+G content between chromosomes within species comes from the U-shape co-variation pattern between chromosome size and C+G content in animals (Figure 9). These findings have enhanced our knowledge of the correlation between C+G content and genome or chromosome size. Further research is required to investigate the molecular mechanisms behind these *trans*- and *cis*-regulations of C+G contents. Potential cellular-level *in-trans* control can include enzymatic regulation, cellular-level gene regulation on transposition, and the influence of substrate concentration. Candidate local *in-cis* factors may include, for example, specific transposition, gene amplification, chromosome number and structural variation.

The dynamics of genome-size variation in many eukaryotes can be largely explained by transposable elements [22]. For example, SINE (short interspersed elements) represents one of the drivers of genome evolution [23]. In maize, 49% to 78% of the genome is made up of retrotransposons (transposons via RNA intermediates) [24]. In wheat, about 90% of the genome consists of repeated sequences and 68% consists of transposable elements [25]. Around 42% of the human genome is made up of retrotransposons, whereas DNA transposons only account for about 2% to 3% [26]. It is possible that *Bytmar1*, a transposon that is rich in GC and is found in the deep-sea hydrothermal crab, encodes three transposase isoforms from a single open reading frame [27]. It follows that the C+G content should be species-dependent. The transposon type may also partly explain why the correlation between C+G content and chromosome size was positive, negative, or very weak depending on the non-mammal animal species considered in this study. Various mammal animals, particularly primates were found to have a negative correlation between C+G content and chromosome size. Does this mean that a *cis*-acting, GC-poor transposon contributed greatly to primate chromosome size? Clearly, more research is required to clarify this matter. It is also very interesting that even though cereals (monocots) were found to have much higher C+G contents than dicot plants, cereals did not show any strong correlation between C+G content and chromosome size in this study. This finding of an animal–plant difference in the correlation between C+G content and chromosome size may stimulate further comparative research on the regulation of base composition in mammalian animals and plants with a view to determining whether C+G content or genome/chromosome size serves as the main driving force during the interactive evolution between the two genome characteristics.

### Somatic Genome Variation of C+G Content

Somatic genome variation (i.e., somagenetic variation) may have partly contributed to the C+G content difference we found between species and between chromosomes in the same species, in our analysis of animals and plants. In flax [28], [29] and certain vertebrates [30]–[32], studies have shown that the genome varies during development and in response to the environment [28], [29], [33], [34]. Viral infection can also promote genome rearrangement in plants [35]. Even in the same plant species (*Nicotiana sylvestris*), somagenetic variation can be stable [36] or unstable over sexual generations [37] depending on the type of genome variation that occurs. Therefore, the genome sequences we analyzed only provide a snapshot of some genomes; at least minor variation in C+G contents is expected if other DNA samples from the same species are sequenced.

In addition, it is known that genome abnormalities and mutation directions can be influenced greatly by dNTP concentrations and non-proportional disturbance in the dNTP pool as well as by temperature range conditions [38]–[41]. DNA polymerase stability can be influenced by environmental factors such as UV radiation [42]. Deamination of DNA can also change base composition [43]. These influences may at least partly explain the C+G content variation and the patterns of correlation between C+G content and genome size in archaeans and bacteria. Further research is required to clarify whether this non-transposition influence also exists in eukaryotes.

### Speciation, Progressive Changes and Sudden Changes

It is interested to note that the large groups of living organisms have distinct C+G contents or correlations between C+G content and chromosome size (Figure 1). According to Darwin’s theory [44], evolution is “a slow and gradual process–one species first giving rise to two or three varieties, these being slowly converted into species,” and a consequence of natural selection is “Divergence of Character and the Extinction of less-improved forms.” The C+G contents analyzed in this study of living organisms were therefore likely favoured and preserved during evolution, and the species with intermediate C+G contents likely became extinct. Natural selection undoubtedly played an important role in determining the C+G contents found in living organisms, and the extinction of some unfit species contributed to the gaps between the large groups existing today (Figure 1). However, some large groups such as the mammal subkingdom have a very long history (250 million years) [45], but all the sequenced genomes still have very similar C+G contents (Figure 1). This may indicate that the evolution path from variety to species may not be as smooth as Darwin believed. A sudden major gene mutation resulting from a process involving transposition, deamination, dNTP pool balance, or DNA polymerase might accelerate the process. The rate of change slows when the new species or group is nearly equilibrated in terms of cellular function, biochemical resource usage, body part cooperation, and adaptation to the environment, and therefore the C+G contents are largely similar among the species of the new group. Both progressive changes and sudden changes contribute to speciation. Further research at the molecular level is required to verify why the C+G contents differ so much between large groups of living organisms.

### C+G Contents in Whole Genomes and Specific Function Units

Although the genomic C+G content varies greatly among kingdoms, subkingdoms, and species (Table 3), the C+G contents in certain functional units were often found to be less variable. The longest coding sequences-exons in various vertebrates and prokaryotes tended to be GC-rich [14]. The RNA poly(A) tail starting position of poly(A) site is usually an adenosine in every species of microorganisms, plants and animals studied to date [16]. Uracil (U) content was found to be higher in the mRNA 3′ untranslated region (3′UTR) than in the pre-mRNA 3′ cleaved-off region (3′COR), and always higher than that of the whole genome in every subkingdom of plants and animals [46]. The genomic T content appears to be too low in monocotyledonous genomes and must be enriched to a certain degree to permit the 3′COR and 3′UTR to function properly in monocotyledonous cells [46]. The functional units, such as 3′COR and 3′UTR, on genome are found to be relatively stable while the whole genomes diverge much faster in terms of the base composition [46]. Further research is required to understand the roles of genomic base composition in evolution of living organisms.

This study analyzed C+G contents and their correlations with chromosome size in different kingdoms of living organisms. Positive, negative, or insignificant correlations were found depending on the kingdom, large phylogenetic group or species considered. Certain co-variation patterns between chromosome-size and C+G content were conserved between species within groups of animals and plants. This suggests that different molecular and evolutionary mechanisms, e.g., genetic control by both *trans*- and *cis*-factors, are responsible for the different patterns of C+G content and genome or chromosome size in different kingdoms, groups, and species.

## Methods

### Genome and Chromosome Sequences

The completely sequenced genomes and various incomplete but assembled genomes in the GenBank, RefSeq, EMBL, DDBJ, and PDB databases were downloaded from the National Center for Biotechnology Information (NCBI) website (http://www.ncbi.nlm.nih.gov). For bacterial genomes, because of limitations in terms of computational power, only the 508 most recently sequenced genomes (non-redundant ones) were analyzed. For archaeans and bacteria, only the complete sequences of the genomes were used. For species in other kingdoms, the chromosomes were essentially described either as complete sequences or as whole-genome shotgun sequences grouped into specific chromosomes. Although the human genome sequence had not yet been completed and did not meet these criteria, it was included in this analysis because of its obvious importance and its interest for readers. If the same strain was sequenced independently by different research groups and there was more than one genome sequence in the NCBI, we randomly chose one of the sequences if sequence length was identical. The genome and chromosome ID list is provided in Table S1.

### C+G Content Calculation

The genome or chromosome sizes and the base compositions were calculated using PERL scripts. The base composition characteristics included the percentages of A, C, G, T, C+G, and A+T, and the (A+T)/(C+G) ratio. Genome base composition was calculated from the overall content of each specific base in the overall amount of bases in that genome. The average chromosome base composition was calculated by taking the average value of all the chromosomes after calculating the base composition of each chromosomes or large scaffold. The genome C+G content and the average chromosome C+G content were very similar but often not exactly the same.

### Correlation Analysis

The genome size and C+G content were plotted first to see whether there was a linear correlation. If these plots did not show a linear relationship between the C+G content and genome or chromosome size, Pearson correlation analysis and linear regression analysis were not used as they would likely not be suitable. In such a case, we used Spearman correlation analysis to test for monotonic relationship. The Spearman correlation analysis was conducted using the CORREL function in the Excel 2010 data analysis package after ranking the data from the smallest to the largest. The Spearman R values along with the p-values were calculated and verified using the COR.TEST function in the statistical software of R program (http://www.r-project.org/). The p-values were computed using the algorithm AS 89 for *n <1290* and exact = TRUE; otherwise, the asymptotic *t* approximation was used [47], [48]. Probability plots of genome size and C+G content were created to examine the type of distribution (not for correlation analysis), and outliers were identified using the regression function in the Excel 2010 data analysis package.

Calculations and statistical analysis were carried out mainly at the first three of the following levels: 1) genome-level comparison of kingdoms and large groups based on the genome sizes and C+G contents of the genomes; 2) chromosome-level comparison of kingdoms and large groups; 3) chromosome-level analysis within each group and species; and 4) chromosome-type-level analysis for some archaean species, because three of them had one larger chromosome and one smaller chromosome.

We ranked the strength of the Spearman correlation R using the grouping by Swinscow and Campbell [41]: R  = 0.0–0.19 very weak (which we called it “no detectable correlation or “no correlation”), 0.2–0.39 weak, 0.40–0.59 moderate, 0.6–0.79 strong and 0.8–1 very strong. However, we used this ranking of moderate, strong, and very strong R values only if the R values were significantly different from zero (P<0.05).

## Supporting Information

Figure S1 **The chromosome C+G contents in** ***Gallus gallus*** **.** Note that, on average, C+G content and chromosome size are negatively correlated in this species, but four chromosomes behaved differently.
(DOCX)

Table S1 **Genome and chromosome ID list.**
(XLSX)
