# Supplementary material for: Variation, Evolution, and Correlation Analysis of C+G Content and Genome or Chromosome Size in Different Kingdoms and Phyla
Source: PLoS One. 2014 Feb 13;9(2):e88339. doi: 10.1371/journal.pone.0088339 (PMC3923770; doi:10.1371/journal.pone.0088339)
Supplement: Figure S1 — The chromosome C+G contents in Gallus gallus . Note that, on average, C+G content and chromosome size are negatively correlated in this species, but four chromosomes behaved differently. (DOCX) [file pone.0088339.s001.docx]

**Figure S1. The chromosome C+G contents in *Gallus gallus*.** Note that, on average, C+G content and chromosome size are negatively correlated in this species, but four chromosomes behaved differently (red).
